# Bioprinted Four-Cell-Type Lung Model for Viral Infection Studies Under Air–Liquid Interface Conditions

**DOI:** 10.3390/ijms26125543

**Published:** 2025-06-10

**Authors:** Johanna Berg, Julian Heinze, Daniela Niemeyer, Josefin Hellgren, Himjyot Jaiswal, Anna Löwa, Andreas Hocke, Itedale Namro, Christian Drosten, Jens Kurreck, Beatrice Tolksdorf

**Affiliations:** 1 Department of Applied Biochemistry, Institute of Biotechnology, Technische Universität Berlin, 10623 Berlin, Germanyjens.kurreck@tu-berlin.de (J.K.); 2 German Center for Infection Research (DZIF), Charitéplatz 1, 10117 Berlin, Germany; 3 Institute of Virology, Charité-Universitätsmedizin Berlin, Corporate Member of Freie Universität Berlin and Humboldt-Universität zu Berlin, 10117 Berlin, Germany; 4 Cellink, Långfilsgatan 1-7, 412 77 Gothenburg, Sweden; 5 Department of Infectious Diseases, Respiratory Medicine and Critical Care, Charité-Universitätsmedizin Berlin, Corporate Member of Freie Universität Berlin and Humboldt-Universität zu Berlin, 10117 Berlin, Germany

**Keywords:** bioprinting, human lung model, air–liquid interface culture, SARS-CoV-2, influenza A virus

## Abstract

Viral lung infections are a never-ending threat to public health due to the emergence of new variants and their seasonal nature. While vaccines offer some protection, the need for effective antiviral drugs remains high. The existing research methods using 2D cell culture and animal models have their limitations. Human cell-based tissue engineering approaches hold great promise for bridging this gap. Here, we describe a microextrusion bioprinting approach to generate three-dimensional (3D) lung models composed of four cell types: endothelial cells, primary fibroblasts, macrophage cells, and epithelial cells. A549 and Calu-3 cells were selected as epithelial cells to simulate the cells of the lower and upper respiratory tract, respectively. Cells were bioprinted in a hydrogel consisting of alginate, gelatin, hyaluronic acid, collagen, and laminin-521. The models were cultured under air–liquid interface (ALI) conditions to further enhance their physiological relevance as lung cells. Their viability, metabolic activity, and expression of specific cell markers were analyzed during long-term culture for 21 days. The constructs were successfully infected with both a seasonal influenza A virus (IAV) and the severe acute respiratory syndrome coronavirus 2 (SARS-CoV-2) omicron variant, demonstrating their potential for studying diverse viral infections.

## 1. Introduction

Viral infections of the lungs continue to represent a persistent threat and burden to the general public. The threat is posed by both emerging viral variants and seasonal viruses [1]. Although vaccination strategies have been developed, for some viruses, they are challenged by the constant emergence of novel viral variants or subtypes [2,3]. In light of the general risk of viruses evading the human immune system and the continuous emergence of new variants, there is an urgent need for the development of therapeutic strategies and drugs. Despite the success of approved therapeutics or the accelerated approval of vaccines in the case of the severe acute respiratory syndrome coronavirus 2 (SARS-CoV-2) in lowering the prevalence of viral infections, there remains a need to treat those who have been afflicted [4,5]. It is therefore necessary to have rapidly available, reliable model systems for efficient drug testing.

Two-dimensional (2D) cell cultures or suspension cultures are very efficient, especially for high-throughput applications, and have become a useful tool in viral research [6]. However, monolayer cell cultures lack the tissue architecture and organization that are characteristic of native tissues. Thus, they lack the three-dimensional (3D) cell–cell contacts and interactions with different cell types that are present in the human lung [7,8]. As a result, animal models have become an important tool in preclinical research, providing structural and cellular complexity as well as the ability to study systemic effects. However, in addition to ethical concerns, there are also scientific differences. Many animal models are difficult to infect with viruses that have a human tropism, requiring the use of adapted strains [9]. This, together with limitations in reproducing human lung physiology, leads to poor translatability in predicting human responses. The predictability of animal models for humans is often limited, and species–specific differences very often mean that the findings in animal studies cannot be confirmed in clinical trials [10,11,12,13]. In addition, animal models are laborious and time-consuming compared to most in vitro models, which limits their applicability and throughput for screening approaches such as those needed in drug development.

The gap between 2D cultured cells, animal models, and human patients can be bridged or reduced by human cell-based tissue engineering approaches. These human cell-based models can closely mimic the physiological conditions found in the human body, making them more reliable for studying disease mechanisms and drug responses. In clinical drug efficacy screening, these models can be used to pre-select therapeutics for animal testing, or even replace animal testing if they are sufficiently advanced [14,15,16,17]. One of the technologies used to create these 3D cell cultures is bioprinting. It aims to reproduce the complexity of tissues in vitro, by generating a structurally defined, cell-laden matrix in which the cells are spatially distributed or arranged in a pre-designed 3D environment [18]. By implementing bioprinting, preclinical human in vitro models can be reproducibly generated in the quantities required, combining the benefits of multiple human cells in a spatially arranged matrix [19,20,21,22,23]. The culture conditions of these models after printing strongly influence the cellular behavior. With respect to in vitro lung models, as described here, the culture of the bioprinted models under air–liquid interface (ALI) conditions is an important step to physiologically mimic the natural environment of the human lung epithelium [7,24].

Recent advances in 3D lung model development include organoids, lung-on-a-chip systems, and hydrogel-based cultures [14]. For example, Zou et al. created a bioprinted 3D lung cancer model consisting of A549 cells to compare differential genes and functions of 2D and 3D lung cancer cells using RNA-sequencing techniques [25]. Sanchez-Guzman et al. developed an ALI-cultivated 3D model of the human bronchial epithelium using the Calu-3 cell line to characterize the long-term evolution of the epithelial cell secretome via quantitative mass spectrometry [26]. However, many models lack the ability to combine multiple relevant lung cell types or replicate native tissue structure under ALI conditions.

Here, we present a novel model that addresses the key limitations of the existing models, including submerged cultivation conditions and limited cell type complexity. By combining micro-extrusion bioprinting using three different print heads, we generated a lung model consisting of four cell types: endothelial cells, fibroblasts, macrophage-like cells, and epithelial cells. The lung model represents an advancement over our previously published grid-based model [19], which exhibited deficiencies in terms of ALI compatibility and the absence of an endothelial layer. The new model consists of a printed base layer of endothelial-like HMEC-1 cells, a second printed layer of primary lung fibroblasts and macrophage-like THP-1 cells, followed by seven printed layers of pure bioink that act as a rim. The epithelial cells were seeded into the round open mold, and the constructs were cultured under ALI conditions. The four cell types were selected based on their relevance to the key lung compartments. HMEC-1 cells represent the vascular endothelium, primary lung fibroblasts provide structural integrity, THP-1-derived macrophages emulate innate immune function, and A549 and Calu-3 cells model alveolar and bronchial epithelial surfaces, respectively. Despite their inability to perfectly represent in vivo conditions, the immortalized cell lines were selected based on their well-characterized growth behavior, robust nature, ease of culture, and reproducibility.

The models were infected with either the human seasonal influenza A virus (IAV) strain Panama/1999/2007 (H3N2) or the SARS-CoV-2 omicron BA.2 virus strain hCoV-19/Germany/SH-ChVir26729_2/2022. The influenza virus replicated efficiently in the models containing Calu-3 or A549 cells as epithelial cells, whereas the coronavirus replicated in the Calu-3 cell model.

Taken together, the new lung model integrates four distinct cell types and supports infection under ALI conditions, marking a novel advance in bioprinted lung models.

## 2. Results

Previously, we developed and tested a human lung model consisting of a bioprinted base layer of primary human lung fibroblasts and macrophage-like THP-1 cells. Alveolar epithelial A549 cells were printed on this base as a second layer. The cells were embedded in a hydrogel consisting of alginate, gelatine, and collagen. These constructs were maintained in long-term culture and could be infected with IAV [19].

### 2.1. Generation of an Improved Bioprinted Four-Cell-Type Lung Model

**Figure 1:**
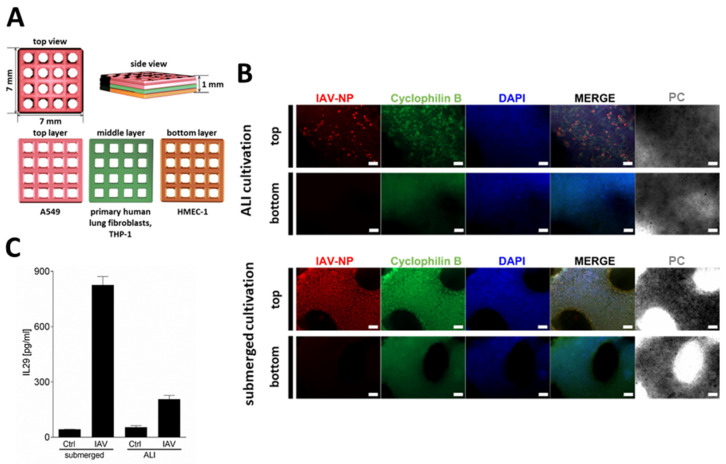
Bioprinted multi-cell-type lung model. (**A**) Schematic representation of the multi-layered lung model consisting of a printed bottom layer of endothelial-like HMEC-1 cells, a printed middle layer of primary lung fibroblasts and macrophage-like THP-1 cells, followed by a printed top layer of alveolar type II-like A549 cells. (**B**) Staining of whole lung models that were infected with 1 × 10^6^ pfu/mL of IAV 14 days after the start of ALI or submerged culture. Models were fixed 24 h after infection, immunochemically stained for IAV nucleoprotein (red) and cyclophilin B (green), cleared, and analyzed via fluorescence microscopy. Nuclear counterstaining was performed with DAPI (blue). Scale bar: 200 μm. (**C**) Centrifuged supernatants 24 h after IAV infection were assayed for IL-29 release via enzyme-linked immunosorbent assay. Results are presented as mean ± standard deviation of three independent experiments.

In this study, the model was improved to better reflect the physiological conditions in the human lung by adding a bottom layer of endothelial-like HMEC-1 cells and by culturing the models under ALI conditions. Initially, the hypothesis was tested as to whether culturing a bioprinted lung model under ALI conditions may influence the outcome of an IAV infection. Consequently, standard grid models were produced via pneumatic micro-extrusion, containing a base layer of HMEC-1 cells and a second layer containing human lung fibroblasts, interspersed with THP-1 and HMEC-1 cells. A third layer containing A549 cells was then printed on top. The different layers and the corresponding holes in the layers of the grid model are shown in Figure 1A. The printed models were cultured for three days under submerged conditions, during which time they were permitted to recuperate following the printing process. Subsequently, the models were transferred into transwell inserts for ALI culture. The layer comprising solely HMEC-1 cells and the one containing fibroblasts, in conjunction with HMEC-1 and THP-1 cells, were placed in the liquid phase, while the epithelial A549 cell layer was situated in the gas phase. The constructs were cultured for 14 days prior to infection with the IAV strain H3N2 Pan/99 for 24 h.

An even cell distribution in the grid models was confirmed via DAPI staining (Figure 1B). The staining of the models for IAV-NP demonstrated that submerged culture resulted in a more homogeneous distribution of the infection throughout the epithelial cells (Figure 1B). In contrast, the ALI-cultured epithelial cells exhibited a more spot-like, clustered infection pattern (Figure 1B). This pattern was characterized by the presence of areas of IAV-infected A549 cells, interspersed with areas that were free of IV-NP signal. Furthermore, the release of IL-29, the main interferon secreted by IAV-infected alveolar type II epithelial cells [27], was quantified via ELISA (Figure 1C). The submerged models released significantly more IL-29 compared to the ALI-cultured models, which could result from the lower number of infected epithelial cells in the latter.

For the infection of the ALI-cultured models, IAV was only pipetted dropwise in a total amount of 20 µL onto the epithelial cells, so an excessive amount of liquid was not present on the surface, but we had no assurance that some of the fluid did not drip off the stack. Furthermore, it was challenging to ascertain that the epithelial cells remained within the gas phase throughout the entire culture period. The transwell inserts and the model are not transparent, and if the medium level is elevated only slightly, the epithelial cell layer may have been partially submerged.

**Figure 2:**
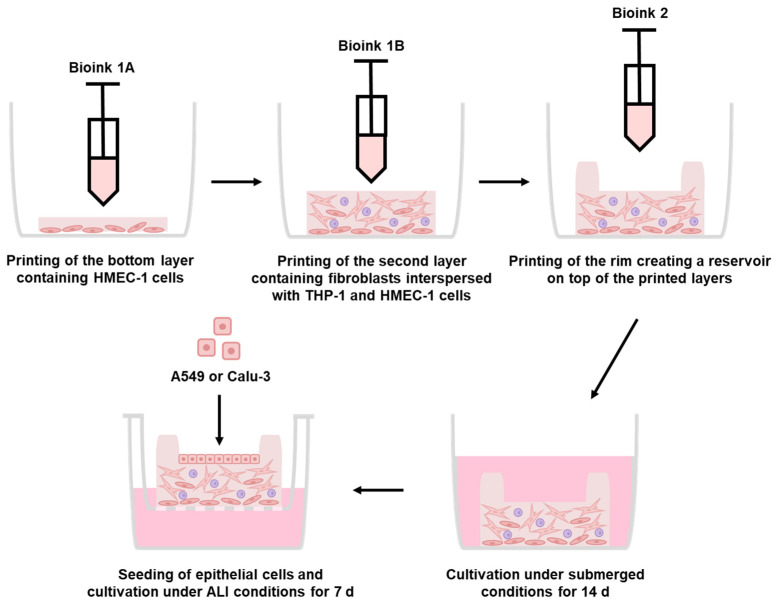
Generation of the improved bioprinted multi-cell type lung model via extrusion printing. Schematic representation of the printing process of the multi-layered lung model consisting of a printed base layer of endothelial-like HMEC-1 cells, a second printed layer of primary lung fibroblasts and macrophage-like THP-1 cells, followed by seven printed layers of pure bioink acting as a rim. Epithelial cells can be seeded into the round open mold and cultured under ALI conditions. The full experimental procedure can be found in the graphical abstract.

To improve the settings and generate an epithelial (mono)layer, the model was redesigned. The model retained a base layer comprising HMEC-1 cells and a second layer comprising lung fibroblasts interspersed with THP-1 and HMEC-1 cells (Figure 2).

**Figure 3:**
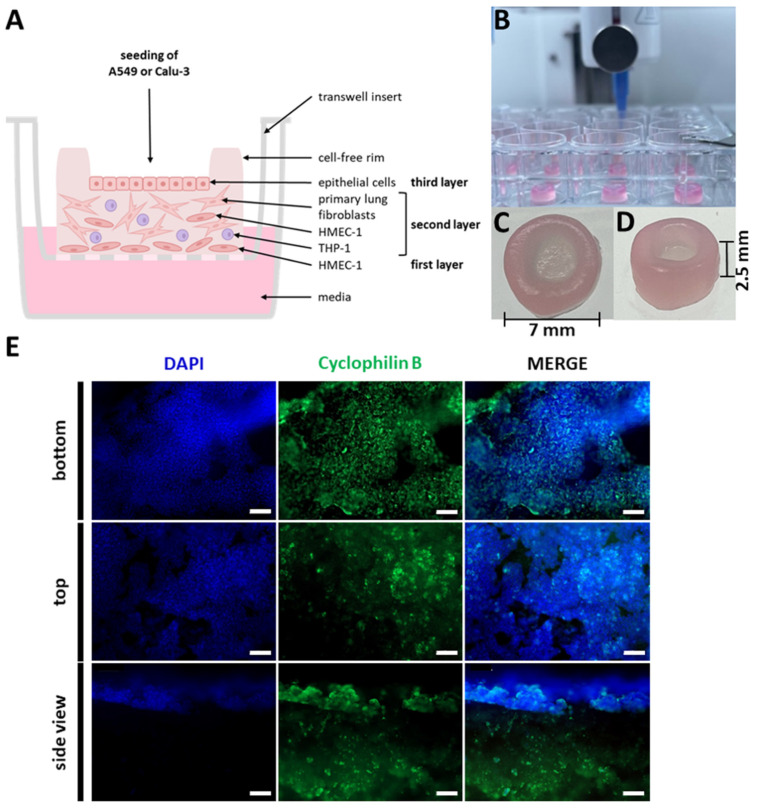
Improved bioprinted four-cell-type lung model. (**A**) Schematic representation of the multi-layered lung model consisting of a printed base layer of endothelial-like HMEC-1 cells, a second printed layer of primary lung fibroblasts and macrophage-like THP-1 cells, followed by seven printed layers of pure bioink acting as a rim. Epithelial cells can be seeded into the round open mold and cultured under ALI conditions, while the lower layers of the model (containing HMEC-1, fibroblasts, and THP-1) are partially in contact with the medium. (**B**) Bioprinting of the lung models via extrusion. Photographs of the bioprinted model in top (**C**) and side view (**D**). (**E**) Staining of a whole lung model seeded with A549 cells seven days after the start of ALI culture. The model was fixed, immunochemically stained against cyclophilin B (green), cleared, and analyzed via fluorescence microscopy. Nuclear counterstaining was performed with DAPI (blue). Scale bar: 100 μm.

A seven-layer high boundary/rim structure was then printed on top of these layers (2.5 mm height), creating a reservoir on top of the printed layers that could be used to hold small amounts of liquids, with a maximum capacity of 25 µL (Figure 3A). The model was designed to be 7 mm in diameter and 2.5 mm in height to fit into standard 24-well plates, allow transfer to the ALI culture, and enable media changes and sample collection.

Following printing (Figure 3B), epithelial cells were seeded into the cavity to generate the epithelial layer (Figure 3C,D). By seeding the epithelial cells instead of bioprinting them, it is possible to simulate the upper or lower respiratory tract conditions, or even pathophysiological conditions, without having to laboriously adapt the bioink to the specific requirements of different cell lines. For the subsequent experiments, the A549 cell line was selected to represent the lower respiratory tract, while the Calu-3 cell line was chosen to represent the upper respiratory tract. Furthermore, the rim structure prevented the influx of media, ensuring that the epithelial layer remained permanently in the gas phase, while the lower layers of the model (containing HMEC-1, fibroblasts, and THP-1) were partially in contact with the medium. After the models were printed via extrusion, they were cultured for 14 days under submerged conditions (Figure 2). This allowed the cells to recover and begin spreading following the printing procedure. Subsequently, the models were transferred to transwell inserts, the excess media within the rim structure was removed, and epithelial cells were seeded in a volume of 15 µL. After three days of culture, the excess media was again removed from the inner part of the rim structure, and the models were cultured for seven days under ALI conditions, maintaining the epithelial cells in the gas phase and the lower layers of the model in partial contact with the medium.

### 2.2. Characterization of the Improved Bioprinted Four-Cell-Type Lung Model

In order to visualize the distribution of the cells and determine whether the seeded epithelial cells had attached to the model, the entire printed construct was analyzed using immunofluorescent staining. As an example, the top, bottom, and cross-sectional views of a model seeded with A549 cells that was stained against cyclophilin B in conjunction with nuclear counterstaining using DAPI are presented in Figure 3E. It is evident that there are morphological differences between the cells in the printed and seeded layers. As observed in the top view, the seeded A549 cells have formed a cell-connected layer. Similarly, the HMEC-1 cells in the bottom view have formed a dense structure of connected cells. However, the fibroblast layer is only visible within the side, cross-sectional view, indicating that the cells align themselves uniformly along the horizontal axis. Given that fibroblasts possess a longer spindle-like shape, the probability of observing the nuclei of fibroblasts in the cross section is relatively low.

However, due to the inherent softness of the models, it proved challenging to image their cross sections. To address this technical limitation and to facilitate the staining of cross sections while reducing background fluorescence, the specimens were henceforth first embedded in paraffin before immunohistochemical staining. Furthermore, the number of seeded epithelial cells was adjusted to permit both cell types, A549 and Calu-3, to form a layer within the seven days of ALI culture. To achieve this, 2 × 10^5^ Calu-3 cells were required, whereas only 5×10^4^ A549 cells were needed.

**Figure 4:**
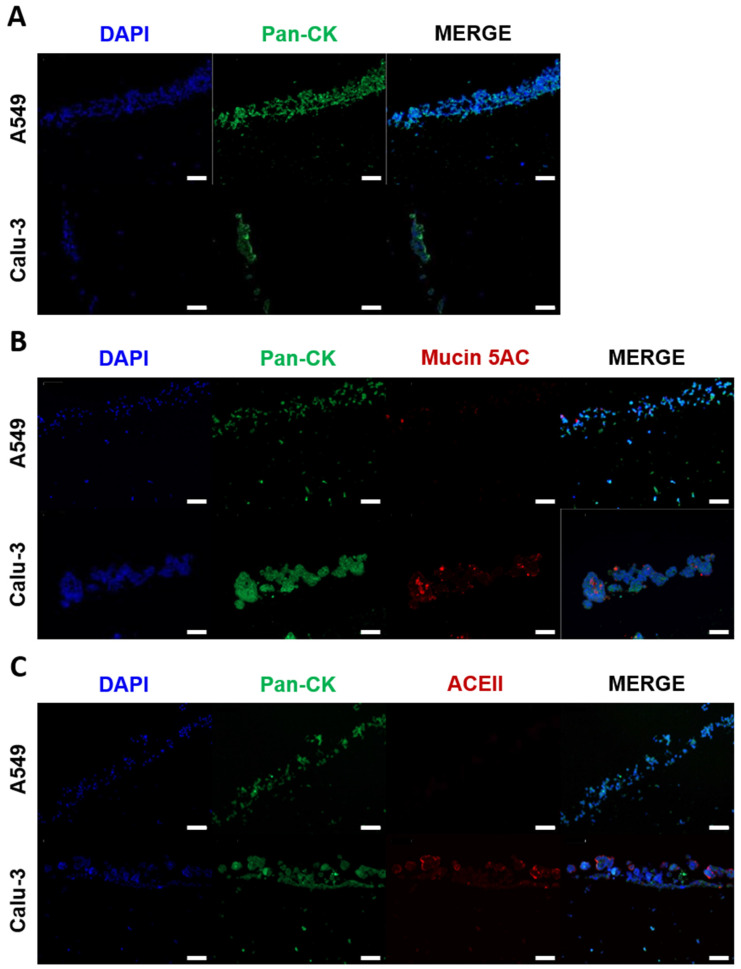
Immunohistochemical analysis of the improved bioprinted multi-cell-type lung model. Differential protein expression and morphology between the printed cells were analyzed seven days after the start of ALI cultivation. Models containing endothelial-like HMEC-1 cells, primary lung fibroblasts, and macrophage-like THP-1 cells seeded with alveolar type II-like A549 cells or bronchial-like Calu-3 cells were fixed, dehydrated, and embedded in paraffin for microtome sectioning. Immunohistochemical staining was performed against (**A**) pan-cytokeratin (green), (**B**) mucin 5AC (red), or (**C**) ACEII (red), and the sections were analyzed via fluorescence microscopy. Nuclear counterstaining was performed with DAPI (blue). Scale bar: 100 μm.

In accordance with the model generation strategy, a specific distribution of the cells could be observed. Models seeded with A549 or Calu-3 cells demonstrated the expression of pan-CK (Figure 4A–C, green channel) in the seeded epithelial layer. However, there were some differences in the arrangement of the cells. The seeded Calu-3 cells clustered into aggregates, which is consistent with the observed characteristics in 2D culture, whereas the A549 cells remained relatively planar.

The mucus layer provides a crucial first barrier to inhaled pathogens that can prevent pathogen invasion and subsequent infection. However, mucus production does not occur to a significant extent in submerged Calu-3 and A549 2D and 3D cultures [28]. To test whether the ALI cultivation induced the expression of mucin, the Calu-3 and A549 seeded models were stained using an antibody against mucin 5AC (green channel) and pan-CK (red channel) after seven days of ALI culture. As illustrated in Figure 4B, seeded Calu-3 cells exhibited a markedly higher expression of mucin 5AC compared to seeded A549 cells. This finding is consistent with the notion that Calu-3 cells exhibit characteristics similar to those of human bronchial submucosal glands, whereas A549 cells are more representative of the distal respiratory tract, which is known to secrete less mucus [28].

The bioprinted models were intended to be used for infection with the SARS-CoV-2 omicron BA.2 virus strain. SARS-CoV-2 is known to enter cells by binding to the host cell receptor ACEII [29]. Therefore, the models were stained using an antibody against ACEII (green channel) and pan-CK (red channel). As illustrated in Figure 4C, Calu-3 cells expressed ACEII, while A549 cells did not exhibit any expression, which is in line with previously published studies [30]. As a result, the A549 cultures were not infected with the SARS-CoV-2 omicron BA.2 virus strain.

**Figure 5:**
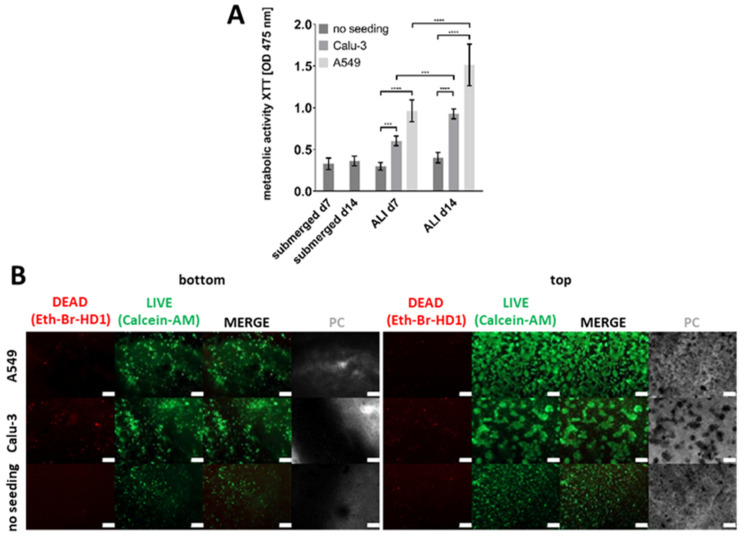
Metabolic activity and cell viability staining of the improved bioprinted four-cell-type lung model. (**A**) Metabolic activity as determined via the tetrazolium hydroxide salt (XTT) assay at the indicated time points after printing or after the start of ALI cultivation, respectively. Models containing endothelial-like HMEC-1 cells, primary lung fibroblasts, and macrophage-like THP-1 cells (no seeding), and models additionally seeded with alveolar type II-like A549 cells or bronchial-like Calu-3 cells were analyzed. The results are presented as the mean ± standard deviation of three independent experiments. The statistical significance was determined via a univariate analysis of variance (one-way ANOVA). *** *p* ≤ 0.01, **** *p* ≤ 0.0001. (**B**) Cell viability staining using calcein-AM (live in green) and ethidium homodimer-1 (dead in red) seven days after the start of ALI culture. Different models, as described above, were analyzed. Scale bar: 200 μm.

To further characterize the models, their metabolic activity was quantified using an XTT assay seven and fourteen days after the start of ALI culture (Figure 5A). The models seeded with A549 cells showed the highest metabolic activity, followed by those seeded with Calu-3 cells, while the unseeded models showed the lowest values. However, direct comparisons between A549 and Calu-3 seeded models are challenging due to the differences in the initial seeding densities required by the slower growth rates of Calu-3 cells compared to A549 cells. As a result, absolute XTT values are not directly comparable between these conditions. Nevertheless, the observed trend of increasing metabolic activity over time in both seeded models, in contrast to the stable values in the unseeded models, indicates continued cell growth and viability in the presence of epithelial cells.

To assess the ratio of living and dead cells and to observe the morphology of the cells, live/dead staining was performed for all three types of models, containing either A549, Calu-3, or no epithelial cells seven days after the start of ALI culture (Figure 5B).

Overall, cell viability assays did not reveal a substantial increase in dead cells over the culture period, neither for models with an epithelial layer nor for models without epithelial cells. However, the morphology of the cells began to change over the course of the cultivation period, and there were notable differences between unseeded models and models containing epithelial cells. The HMEC-1 layer of models lacking epithelial cells exhibited less cell spreading compared to the models including either A549 or Calu-3 cells. The second layer, which contained fibroblasts, could be observed in the models without epithelial cells. A small proportion of cells exhibited red staining, while the majority were green fluorescent, indicating that nearly all the cells within this layer were alive. After printing, fibroblasts initially had a round shape resulting from encapsulation in bioink components. However, at later time points, they also started to change their morphology towards the spindle-like structure characteristic of fibroblasts. The seeded epithelial cells were found to have a high proportion of living cells and only a few dead cells. The morphology of the Calu-3 cells differed from that of the A549 cells, as also observed in 2D culture (Figure S1). The A549 cells formed numerous small clusters of cells, which were distributed over the entire surface of the model, as was previously observed in other studies [19]. In contrast, the Calu-3 cells formed larger cell clusters, which were also distributed over the entire surface. Consequently, the fibroblast layer beneath was still visible in a few regions in both cases.

### 2.3. Infection of the Improved Bioprinted Four-Cell-Type Lung Model

**Figure 6:**
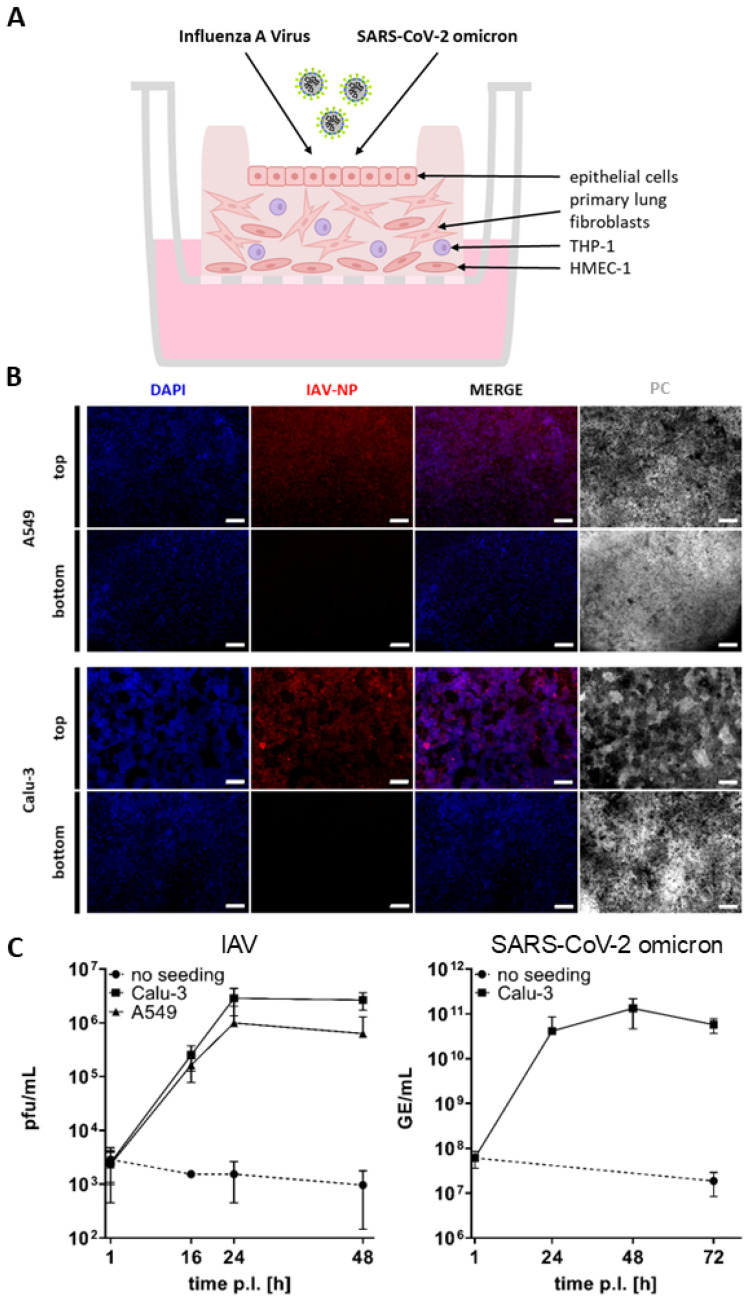
Infection of the improved bioprinted multi-cell-type lung model. (**A**) Schematic representation of the infection procedure. (**B**) Staining of whole lung models infected with IAV at an MOI of 0.01 seven days after the start of ALI culture. Models were fixed 48 h after infection, immunochemically stained for IAV nucleoprotein (red), cleared, and analyzed via fluorescence microscopy. Nuclear counterstaining was performed with DAPI (blue). Scale bar: 100 μm. (**C**) Seven days after the start of ALI culture, lung models seeded with A549 or Calu-3 cells as well as unseeded models were infected with IAV or SARS-CoV-2 omicron at an MOI of 0.01. The infection solution was pipetted into the rim and incubated for 1 h. The models were then rinsed and incubated under ALI conditions. For sampling, media was added inside the rim for 1 h. Samples were collected at 1, 16, 24, and 48 h post-infection for IAV and titrated using a standard plaque assay. For SARS-CoV-2 omicron, Calu-3-seeded models and unseeded controls were sampled at 1, 24, 48, and 72 h post-infection. Genome equivalents per milliliter (GE/mL) were determined via quantitative RT-PCR. Results are shown as mean ± standard deviation of three independent experiments.

Following the thorough characterization of the bioprinted lung model, its susceptibility to infection with the IAV strain Panama/1999/2007 (H3N2) and the SARS-CoV-2 omicron BA.2 virus strain hCoV-19/Germany/SH-ChVir26729_2/2022, which represent the key respiratory viruses, was investigated (Figure 6A).

For infection with IAV, printed models seeded with A549 or Calu-3 cells as well as unseeded models were infected at an MOI of 0.01. To ensure apical infection, the infection solution was pipetted within the rim and incubated for 1 h. The solution was then removed, and the infected models were incubated under ALI conditions after rinsing. To generate a growth curve, samples were collected at 1, 16, 24, and 48 h post-infection and titrated using a standard plaque assay to determine plaque-forming units (pfu)/mL. For sampling, media was added within the rim for 1 h. Subsequently, the samples were fixed, paraffin embedded, and immunohistochemically stained against IAV-NP.

The images and growth curve clearly demonstrate that only the models seeded with A549 or Calu-3 cells were infected with IAV, as indicated by the red fluorescence originating from the viral nucleoprotein (Figure 6B). This is consistent with the observation that the viral titers significantly increased in the models seeded with A549 and Calu-3. Viral titers were found to be slightly higher in the Calu-3 seeded models, reaching 2.87 × 10^6^ pfu/mL after 24 h, while 9.94 × 10^5^ pfu/mL were measured in the A549 seeded models (Figure 6C). In contrast, no viral replication was found in the unseeded models, which only contained endothelial-like HMEC-1 cells, primary lung fibroblasts, and macrophage-like THP-1 cells but no epithelial cells, thus confirming that IAV can only replicate in epithelial cells (Figure 6B,C) [19].

Infection with SARS-CoV-2 omicron was performed on the printed models seeded with ACEII-expressing Calu-3 cells (Figure 4C) and unseeded models at an MOI of 0.01, as described above. A growth curve was generated by isolating viral RNA from the collected samples at 1, 24, 48, and 72 h post-infection. To quantify the extent of viral replication, genome equivalents per milliliter (GE/mL) were determined from the viral RNA extracts via quantitative RT-PCR targeting a conserved region in the SARS-CoV-2 E gene [31,32].

As illustrated in Figure 6C, the SARS-CoV-2 omicron variant exhibited a substantial replication in the models seeded with Calu-3 cells, reaching 4.14 × 10^10^ GE/mL after 24 h and 1.32 × 10^11^ GE/mL after 48 h post-infection. As anticipated, no viral replication was observed in the unseeded models.

Collectively, we have developed a sophisticated lung model composed of four cell types and cultured under ALI conditions, which provides a promising tool for the study of viral infections.

## 3. Discussion

Respiratory infectious diseases present a significant threat due to their capacity to cause widespread outbreaks and severe damage to the lung. The spread and progression of the disease depends on the route of entry and the specific virus. Identifying the cause and mechanisms behind these diseases is crucial for developing effective treatments. This has been recently highlighted in the context of the COVID-19 pandemic, which has demonstrated the emergence of a new virus with a rapid global spread [33]. Although the initial threat posed by the COVID-19 pandemic has diminished with the development of effective vaccines, it has become evident that some of these vaccines have lost part of their efficacy due to the ongoing evolution of SARS-CoV-2 [5,34]. The rapid emergence and evolution of pathogens, therefore, necessitates the swift development of safe and effective treatments in suitable models for studying human-specific infectious viral diseases.

Traditionally, scientists have relied on two main methods to study viral infections: 2D cell cultures and animal models. The 2D cell culture model offers several advantages, including ease of use, consistent results, and affordability [35]. These models have been instrumental in identifying how viruses invade cells, developing drugs that block virus-cell interactions, and discovering potential treatments [36,37,38,39,40,41]. For example, Yang et al. employed the cell line A549 transiently expressing the ACEII receptor to investigate the interaction between the spike protein of SARS-CoV-2 and its cellular receptor ACEII [42]. Similarly, lung epithelial equivalents containing primary cells have been used to model SARS-CoV-2 and influenza infections, providing a physiologically relevant platform to study viral replication and potential treatments [43,44,45,46]. Nevertheless, 2D cell cultures also exhibit inherent limitations. Since cells are cultivated on a flat surface, they lack the spatial organization, complex signaling, and interactions that they experience in living tissues [47,48]. Consequently, the information obtained from 2D cultures may not fully represent the complexity of viral infection in humans.

In addition to 2D cell culture, infectious diseases are often studied in animals, including hamsters, mice, rats, ferrets, and monkeys [49,50,51,52]. Animal models are of great value because they can mimic whole-body responses, including the interactions between organs, systemic inflammation, and different infection routes. However, a lot of animal models are difficult to infect with viruses that have a human tropism and require the use of adapted strains or the humanization of the animals via genetic modification [9,51,53,54,55]. For instance, Hassan et al. created a SARS-CoV-2 mouse model by introducing the human ACEII gene [56]. Another limitation of animal models is that animals and humans possess different genetic compositions, which can result in different symptoms. For instance, mouse models for SARS-CoV-2 do not manifest severe illness comparable to that observed in humans [57].

The difficulties of 2D cell culture and animal models to accurately reflect the intricate mechanisms underlying viral infection, including the mode of viral spread, disease pathogenesis and the temporal progression of disease, highlight the need for the development of novel tools that more closely resemble the human body [58,59]. Human cell-containing 3D engineered tissue models have been developed to address these unmet needs in the field of viral infections. In addition to addressing animal welfare concerns, 3D human models provide more physiologically relevant systems for disease modeling, as well as drug testing, and may therefore be more suitable for studying human-specific viral infections [14,48,60,61]. These models include hydrogel-based 3D cell cultures, organ-on-a-chip, and organoids [62]. They offer several important advantages over traditional methods. For instance, these models replicate the natural structure of human tissues by incorporating multiple cell types within a confined 3D space. This enables a more realistic simulation of how viruses infect and spread within the body [15]. Additionally, compared to animal models, 3D models are well-suited for large-scale studies. They can be produced in high numbers, enabling researchers to perform high-throughput and high-content screening of potential drugs and therapies [63,64].

We have been working on the development of a bioprinted 3D human lung model to study the mechanisms of lung diseases and respiratory infections. The precursor model was generated via micro-extrusion bioprinting a base of primary human lung fibroblasts together with macrophage-like THP-1 cells, onto which alveolar epithelial A549 cells were printed. The cells were embedded in a hydrogel consisting of alginate, gelatin, and collagen [19,20]. Compared to our previously reported bioprinted lung model, the current study introduces several significant advancements. First, in order to more accurately reflect the physiological conditions, hyaluronic acid was included in the hydrogel, and a bottom layer of endothelial cells was added. Second, the new rim-based geometry permits ALI culture and the seeding of the epithelial cells as a layer on top—both of which were not possible with the grid design. A549 and Calu-3 cells were seeded as an epithelial layer. This demonstrates the ability to produce models that mimic the upper or lower respiratory tract to a certain extent. However, to fully reflect the different parts of the respiratory tract, an appropriate cell composition that includes region-specific primary epithelial and supporting cell types would be required. We show that the epithelial layer is essential for successful infection with IAV or SARS-CoV-2. IAV infected both models. As A549 cells did not express the SARS-CoV-2 entry receptor, ACE II, we only used the model for the upper respiratory tract containing Calu-3 cells to study SARS-CoV-2 infection and found the virus to actively replicate in the bioprinted model.

In contrast to 2D in vitro models, where typically a virus induces cellular infection in a single instance, and an excessive amount of the virus is delivered to the cells, 3D engineered tissue models provide a more physiologically relevant environment for studying viral infections. In the context of the global spread of the novel coronavirus SARS-CoV-2, researchers focused on the lung as the first point of infection for the virus [15,42]. Youk et al. developed a 3D model using human lung alveolar type 2 (hAT2) cells, arranged in cyst-like structures within Matrigel. When exposed to SARS-CoV-2, these cells demonstrated increased activity of the genes related to inflammation and antiviral responses [65]. A further study investigated the role of club cells in the distal lung. Salahuddeen et al. created a 3D model of this tissue and found that SARS-CoV-2 viral particles were concentrated in these cells [66]. Si et al. used an organ-on-a-chip platform to study potential treatments. This microfluidic device contains two channels separated by a porous membrane. Human airway and lung cells were cultured on either side of the microfluidic device. The study demonstrated that amodiaquine, an antimalarial drug, effectively prevented infection in this model, while hydroxychloroquine did not [67]. Finally, Lee et al. employed inkjet-based 3D bioprinting to develop a sophisticated 3D ALI culture model composed of epithelium, extracellular matrix, and endothelium that was susceptible to SARS-CoV-2 infection. However, in comparison to the model developed in this study, only one type of epithelial cell line and no immune cells were printed [68]. Overall, research has shown that 3D in vitro models exhibit enhanced sensitivity to viral infection and a pattern of infection more closely resembling that observed in vivo compared to 2D in vitro models [19,69,70,71].

We only infected Calu-3 seeded models with SARS-CoV-2 as we did not detect ACE II expression in the A549 seeded models. However, Sasaki et al. found that ALI culture can induce ACE II expression in A549 cells [72]. Differences in the culture conditions and the duration of ALI culture may account for this difference.

Our infection data furthermore revealed that SARS-CoV-2 replication in Calu-3-based models peaked at 24 h post-infection and remained relatively stable thereafter. This is in contrast to some previous reports, such as Park et al., where viral replication continued to increase over 72 h in 2D culture models [73]. The differences observed between our study and the previous literature may indicate a fundamental divergence in viral replication dynamics between 2D and 3D culture systems. In 3D models, factors such as spatial organization, cell–cell interactions, and tissue-like architecture may influence the viral spread or access to target receptors in ways that differ from monolayer cultures. Further studies directly comparing viral kinetics in 2D and 3D systems may provide additional insight into these mechanisms.

While our study demonstrates that bioprinted lung models using immortalized cell lines can be successfully cultured under ALI conditions to study viral infections, our model has several limitations. For example, the interpretation of cellular responses to infection in this 3D-printed multi-cellular environment requires further investigation. Furthermore, maintaining a stable and functional four-cell construct over time presents challenges, particularly with respect to cell interactions and long-term viability. Although the live/dead staining indicates general cell viability within the construct and in the different layers of seeded and unseeded models, it cannot confirm the survival of specific cell types, such as HMEC-1, THP-1, and primary fibroblasts. However, the successful use of cell-type-specific markers was not feasible in our model, likely due to the cancerous origin of some of the cell lines. In addition, the use of immortalized cell lines rather than primary cells may limit the physiological relevance of our model. For instance, the seeded epithelial cells clustered into aggregates rather than forming a uniform monolayer. This may be due to the cultivation on the gelatin/alginate/HA hydrogel scaffold or the medium used for co-cultivation of the distinct cells in the bioprinted model, which differs from the medium recommended by the ATCC. This aggregation impedes the formation of a functional epithelial barrier, limiting the replication of in vivo cellular behaviors and transepithelial electrical resistance (TEER) measurements. In order to improve the model and address these limitations, primary cells should be used. For example, a recent study demonstrated the potential of human iPSC-derived alveolar organoids to model SARS-CoV-2 infection dynamics and host responses, providing a powerful tool for studying variant-specific effects [74]. In addition, sophisticated lung-on-chip models that integrate primary human epithelial and immune cells allow the precise recapitulation of the respiratory microenvironment, facilitating high-resolution studies of viral pathogenesis and therapeutic interventions [75]. While our model is a step toward greater physiological relevance, future work should aim to incorporate primary cells to further enhance the clinical translatability.

The ability to easily modify the 3D model in regard to infection susceptibility by seeding different epithelial cell types is of great importance, as not only newly emerging viruses, such as SARS-CoV-2, pose a significant threat to global health. Influenza viruses cause a range of common respiratory illnesses, including fever and muscle aches, with a relatively short incubation period. These infections can be serious, particularly for individuals with chronic health conditions, and some IAV strains cause more severe illness than others [76,77]. Given the distinct surface proteins of different influenza viruses, researchers are developing models to study the impact of these variations on infection and disease progression. For instance, Zhou et al. created 3D human airway models to compare the effectiveness of different influenza subtypes in infecting cells. Their findings indicated that viruses that readily infect humans replicated more efficiently than those less transmissible to humans [68]. Another study by Chen et al. examined the effects of influenza (H7N9) on human airway epithelial cells grown in 3D cultures. They found that the replication kinetics differed considerably between traditional 2D cell culture and 3D cell culture. Furthermore, they observed that antiviral drugs demonstrated different efficacies depending on the cell culture method [78]. These findings emphasize the importance of using 3D models for the investigation of influenza virus infections, as they can facilitate a more precise representation of the virus’s behavior within the body and the potential efficacy of pharmaceutical agents.

Even though 3D in vitro models of infectious viral diseases have shown promise, they continue to face challenges in fully capturing the intricate interplay between viruses, multiple organs, and the immune system that occurs during infections in the human body. The combination of different organ models, particularly when incorporating primary cells and implementing dynamic culture conditions, achieved through perfusion techniques, offers a solution [23,79]. This approach would be of particular value in the study of disease development and the evaluation of the effects of drugs, as it allows for a more comprehensive analysis of viral spread and its effects on different organ systems. By studying the viral interactions across multiple organs and the immune system, researchers could gain a deeper understanding of complex infectious diseases and develop more effective treatment strategies.

## 4. Materials and Methods

### 4.1. Cell Culture and Virus Preparation

Human alveolar type II-like cells (A549; CCL-185, ATCC, Manassas, VA, USA) and normal human primary lung fibroblasts (NHLFb; ATCC) were cultured in Dulbecco’s modified Eagle’s medium (DMEM; Biowest, Nuaillé, France) supplemented with 5% fetal calf serum (FCS; c.c.pro, Oberdorla, Germany), 1% L-glutamine (Biowest), and 1% penicillin/streptomycin (P/S; Biowest). Human bronchial-like cells (Calu-3; HTB-55, ATCC) were cultured in DMEM supplemented with 5% FCS, 1% sodium pyruvate (Sigma, Steinheim, Germany), 1% non-essential amino acids (NEAA; Biowest), and 1% P/S. Human endothelial-like cells (HMEC-1; CRL-3243, ATCC) were cultured in MCDB 131 medium (Thermo Fisher Scientific, Waltham, MA, USA) supplemented with 5% FCS, 5% L-glutamine, 1 µg/mL hydrocortisone (Merck, Darmstadt, Germany), 10 ng/mL human epidermal growth factor (hEGF; Merck), and 1% P/S. Human monocytic-like cells (THP-1; DSMZ, Braunschweig, Germany) were cultured in DMEM supplemented with 5% FCS, 1% L-glutamine, 1% NEAA, and 1% P/S. To induce the maturation of monocytic-like THP-1 cells into adherent macrophage-like cells, the models were treated with 200 ng/mL phorbol 12-myristate-13-acetate (PMA; Merck) for 24 h. Subsequently, printed samples were cultured in the medium without PMA for 48 h before infection. Madine Darby canine kidney (MDCK) cells were grown in MEM (Thermo Fisher Scientific) supplemented with 5% FCS, 1% L-glutamine, and 1% P/S.

The seasonal IAV strain Panama/1999/2007 (H3N2) was grown on MDCK cells as described before [80]. Virus stocks were aliquoted, stored at −80 °C and titrated on MDCK cells by using plaque assays. Virus stock production of SARS-CoV-2 omicron BA.2 virus (hCoV-19/Germany/SH-ChVir26729_2/2022) was conducted on Vero E6 cells as described before [81]. Virus stock aliquots were stored at −80 °C and plaque titrated on Vero E6 cells.

### 4.2. Bioink Preparation

Two stock bioinks were prepared (Table 1). For bioink 1 stock, gelatin type A powder (6% *w*/*v*; Sigma), sodium alginate powder (6% *w*/*v*; Sigma), and 0.4% *w*/*v* of hyaluronic acid (HA; Thermo Fisher Scientific) were dissolved in NHLFb media on a magnetic stirrer at 1250 min^−1^, at 37 °C overnight. The hybrid gelatin/alginate/HA hydrogel was mixed with rat tail collagen I (08-115, Merck), laminin-521 (LAM-521; BioLamina, Sundbyberg, Sweden), and the respective cell types, CaSO_4_ (Carl Roth, Karlsruhe, Germany) and NHLFb media. Two final cell-loaded bioinks (1A and 1B) were prepared from stock bioink 1, differing only in the cell types used. The final cell-loaded bioink 1A was composed of 3% *w*/*v* alginate, 3% *w*/*v* gelatin, 0.2% HA, 0.5 mg/mL rat tail collagen I, 500 ng/mL LAM-521, and 0.045 M CaSO_4_ and contained 2.5 × 10^7^ HMEC-1/mL. The second final cell-loaded bioink 1B was also composed of 3% *w*/*v* alginate, 3% *w*/*v* gelatin, 0.2% HA, 0.5 mg/mL rat tail collagen I, 500 ng/mL LAM-521, and 0.045 M CaSO_4_ and contained 2.5 × 10^7^ fibroblasts/mL, 0.5 × 10^7^ THP-1 cells/mL, and 0.5 × 10^7^ HMEC-1/mL. After initial CaSO_4_-driven cross-linking of the alginate (10 min after mixing), bioink 1A and bioink 1B were each loaded into separate print cartridges. For the bioink 2 stock, gelatin type A powder (6% *w*/*v*) and sodium alginate powder (6% *w*/*v*) were dissolved in NHLFb media on a magnetic stirrer at 1250 min^−1^, at 37 °C overnight. The hybrid gelatin/alginate hydrogel was only mixed with CaSO_4_ and NHLFb media. Bioink 2 was processed in the same way as bioinks 1A and 1B, but its final composition was as follows: 3% *w*/*v* alginate, 3% *w*/*v* gelatin, and 45 mM CaSO_4_. After the initial CaSO_4_-driven cross-linking of the alginate (10 min after mixing), bioink 2 was loaded into a separate print cartridge.

### 4.3. Bioprinting

For the bioprinting process, the bioinks were extruded through a 22G needle using the BioX microextrusion printer (Cellink, Gothenburg, Sweden). Bioinks 1A and 1B were extruded at 25–30 kPa (9 mm/s) and bioink 2 at 35–45 kPa (6.5–8 mm/s, 300 ms pre-flow). The 3D construct (diameter: 7 mm, height: 2.5 mm, inner rim volume: ~25 µL) was designed by using the computer-aided design (CAD) software Rhino 7 (Robert McNeel & Associates, Barcelona, Spain). Slicing was performed using HeartOS 1.10.1, which is integrated on the BioX. After printing, the constructs were immersed in 0.1 M CaCl_2_ (Carl Roth) for 10 min to increase alginate gelation and then cultured in an incubator at 37 °C and 5% CO_2_ in a 1:1 mixture of NHLFb and HMEC-1 media. The 1:1 media mixture was supplemented with 10 mM sodium citrate (Merck) and 20 mM CaCl_2_ for 14 days under submerged conditions.

### 4.4. Seeding of Epithelial Cells

After 14 days of culture, the printed constructs were transferred to transwell inserts and corresponding 24-well carrier plates (0.4 MY, Thermo Fisher Scientific) (Figure 2 and Figure 3A,B). All the access media within the rim structure was removed, a defined number of each epithelial cell type was resuspended in a 1:1 mixture of media and LAM-521 and carefully transferred into the rim structure. A total volume of 15 µL was transferred into each rim structure containing either 50,000 A549 cells/model or 250,000 Calu-3 cells/model. Controls without epithelial cells (referred to as unseeded) were covered with a 1:1 mixture of media and LAM-521 without cells. The carrier plate was filled with the 3D culture media mixture described in Section 4.3 until the media level reached the lower parts of the model containing the endothelial cells, fibroblasts, and THP-1 cells, but not the epithelial cells. The rim structure prevented media from entering the free space within the rim structure where the epithelial cells were located. The models were cultured for 72 h (37 °C, 5% CO_2_). During this time, epithelial cells were allowed to attach to the surface of the printed model, and the access media was carefully removed. The models were then cultured for a further seven days (37 °C, 5% CO_2_). During this time, half of the media in the carrier plates of each well was changed every 48 h, leaving the epithelial part within the rim without media.

### 4.5. Cell Viability

The metabolic activity of the bioprinted cells was determined seven days after the start of ALI culture using the tetrazolium hydroxide salt (XTT) assay (AppliChem, Darmstadt, Germany) according to the manufacturer’s instructions. Briefly, printed cell-loaded constructs were cultured under ALI conditions (37 °C, 5% CO_2_) for seven days. For the XTT assay, RPMI medium w/o phenol red was used to minimize the background absorbance. XTT reagent (1 mg/mL) and 100 µM phenazine methosulphate (PMS; Thermo Fisher Scientific) were diluted in RPMI w/o phenol red (Biowest), added to the samples, and incubated submerged for 4 h (37 °C, 5% CO_2_). The absorbance of the resulting solution was measured spectrophotometrically at A475 nm (TriStar Multimode Reader LB942, Berthold Technologies, Bad Wildbad, Germany) with a reference of A680 nm. Cell-loaded constructs incubated in culture medium supplemented with 10% Triton-X-100 (Carl Roth) were used as a lysis control. Values were normalized to the lysis control.

For cell viability assay (Thermo Fisher Scientific), 3D-printed samples were stained with 2 µM calcein-AM and 4 mM ethidium homodimer-1 diluted in RPMI w/o phenol red for 20 min (37 °C, 5% CO_2_, submerged). The samples were analyzed via fluorescence microscopy (Zeiss Observer Z1 microscope; Zeiss, Jena, Germany).

### 4.6. Viral Replication Assay

All the influenza infection experiments were performed under biosafety level 2 (BSL-2) conditions at the Technische Universität Berlin, Germany. To ensure apical infection, we adapted previously published protocols [82,83]. For viral infection, the seasonal IAV strain Panama/1999/2007 (H3N2) stock was diluted to the desired MOI of 0.01 in DMEM supplemented with 2% FCS, 1% L-glutamine, 1% P/S, and 0.2 mg/mL TPCK-trypsin (Thermo Fisher Scientific), which was used as the infection medium. The surface within the rim of the bioprinted samples was washed with HBSS (Biowest) and incubated with the infection solution for 1 h at room temperature to enhance the viral attachment. The solution was then removed, the cells were rinsed twice with HBSS, and the infected models were incubated for 48 h at 37 °C and 5% CO_2_ under ALI conditions. Samples were collected at 1, 16, 24, and 48 h post-infection and titrated on MDCK cells using a standard plaque assay to determine the infectious virus yield. For sampling, 20 uL of infection medium was added within the rim for 1 h.

All the SARS-CoV infection experiments were performed under biosafety level 3 (BSL-3) conditions with enhanced respiratory personal protection equipment at the Charité-Universitätsmedizin Berlin, Germany. For viral infection, the SARS-CoV-2 omicron BA.2 virus stock (hCoV-19/Germany/SH-ChVir26729_2/2022) was diluted to the desired MOI of 0.01 in OptiPRO SFM (Thermo Fisher Scientific) serum-free medium, which was used as the infection medium. Calu-3 cells within the bioprinted model were washed with HBSS and incubated with the infection solution under physiological conditions for 1 h at 37 °C and 5% CO_2_. After 1 h, the virus dilutions were removed, the cells were rinsed twice with HBSS, and the infected models were incubated for 48 h at 37 °C and 5% CO_2_ under ALI conditions. Samples were collected at 1, 24, 48, and 72 h post-infection by adding 20 uL of infection medium within the rim for 1 h. For the isolation of viral RNA, 15 μL of culture supernatant was diluted in 300 μL of MagNA Pure 96 external lysis buffer (Roche, Penzberg, Germany). All the samples were heat-inactivated at 70 °C for 10 min prior to export from the BSL-3. The isolation and purification of viral RNA was performed using the MagNA Pure 96 System (Roche) according to the manufacturer’s recommendations. Viral RNA was quantified using real-time RT-PCR (E gene assay) as previously described [31,32].

### 4.7. ELISA Assay

Cell-released interleukin 29 (IL-29/IFN λ1) was quantified in the culture supernatant using the Human IL-29 ELISA Ready-SET-Go! Enzyme-Linked Immunoabsorbent Assay (ELISA) kit (Thermo Fisher Scientific). This was performed according to the manufacturer’s instructions at an absorbance of A450 nm with a reference of A620 nm (Tristar 5 multimode reader, Berthold Technologies).

### 4.8. Immunostaining

Bioprinted specimens were fixed in 3.7% formalin (Carl Roth) overnight at 4 °C and processed using a commercially available tissue clearing kit (Abcam, Cambridge, UK) according to the manufacturer’s instructions. Shortly, samples were blocked in 5% anti-goat serum (Sigma) in HBSS for 1 h and incubated overnight at 4 °C with the appropriate primary antibodies against cyclophilin B (1:400, ab16045, Abcam) and IAV nucleoprotein (IAV-NP; 1:250, ab20343, Abcam) in staining solution (HBSS with 1% BSA (Carl Roth) and 0.05% Tween 20 (Carl Roth)). The samples were then washed three times with HBSS and incubated with appropriate Alexa 546- and Alexa 488-conjugated secondary antibodies (1:2000, Thermo Fisher Scientific) in staining solution for 2 h at room temperature. After incubation, the samples were washed twice with HBSS, stained with 4′,6-diamidino-2-phenylindole (DAPI; 1 µg/mL, Thermo Fisher Scientific) in staining solution for 30 min to visualize the nuclei, and then washed again with HBSS. For imaging, the samples were finally mounted in clearing buffer II (Abcam) in a 3.5 mm silicon imaging chamber (Abcam) and analyzed via fluorescence microscopy (Zeiss Observer Z1 microscope).

### 4.9. Immunohistochemical Staining

For immunofluorescence staining, the constructs were fixed in 3.7% formalin for 60 min and washed three times with HBSS. The constructs were then dehydrated and embedded in paraffin for microtome sectioning. Sections were cut on a microtome (pfm Rotary 3004 M, pfm medical, Cologne, Germany) at a thickness of 10 µm. After deparaffinization and rehydration, antigen retrieval was performed by incubating the samples in citrate buffer (pH 6.0, Carl Roth) at 95 °C for 30 min, followed by gradual cooling to room temperature. The samples were then washed three times with HBSS, blocked with 5% anti-goat serum in HBSS for 1 h and incubated overnight at 4 °C with the appropriate primary antibodies against pan-cytokeratin (pan-CK; 1:100, ab234297, Abcam), mucin 5AC (1:100, ab3649, Abcam), angiotensin-converting enzyme 2 (ACEII; 1:100, MAB933, R&D Systems, MN, USA), and IAV-NP (1:750, Abcam) at 4 °C overnight. Afterwards, the samples were washed three times with HBSS and incubated with appropriate Alexa 546- and Alexa 488-conjugated secondary antibodies (1:1000) in staining solution for 2 h at room temperature. For nuclear counterstaining, the samples were incubated with 1 µg/mL DAPI in staining solution for 30 min. The stained samples were analyzed via fluorescence microscopy (Zeiss Observer Z1 microscope).

### 4.10. Statistical Analyses

Statistical significance was analyzed via one-way ANOVA or Student’s t-test using GraphPad Prism 8 software (La Jolla, CA, USA). All the data were collected in triplicate, in at least three independent experiments and are presented as mean  ±  standard deviation (SD). Statistical significance is indicated as * *p*  ≤  0.05, ** *p*  ≤  0.01, *** *p*  ≤  0.001, **** *p*  ≤  0.0001.

## 5. Conclusions

In conclusion, this study reports the development of an advanced bioprinted 3D human lung model incorporating four key cell types: endothelial cells, primary fibroblasts, macrophage cells, and epithelial cells. This model, cultured under ALI conditions, mimics certain aspects of the physiological environment of the human lung and can be adapted to address scientific questions. The use of appropriate cell types allowed partial replication of upper and lower respiratory tract characteristics. The results of this study suggest that bioprinted human lung models using immortalized human cell lines hold potential to bridge the gap between traditional 2D cell cultures, animal models, and human patients. The bioprinted lung models demonstrated the ability to replicate infection patterns of both seasonal IAV and the SARS-CoV-2 omicron variant, indicating their potential as a robust and ethical tool for studying respiratory viral infections and antiviral drugs. However, while these constructs offer advantages over traditional 2D cultures and animal models, the incorporation of primary human cells would further enhance their clinical translatability. Future studies should focus on integrating such cells to develop even more representative lung models.

## Figures and Tables

**Table 1:** Composition of bioinks.

Ingredients	Bioink 1A	Bioink 1B	Bioink 2 (Rim)
Alginate [% *w*/*v*]	3	3	3
Gelatin [% *w*/*v*]	3	3	3
CaSO_4_ [mM]	45	45	45
HA [% *w*/*v*]	0.2	0.2	-
Collagen I [mg/mL]	0.5	0.5	-
LAM-521 [µg/mL]	10	10	-
HMEC-1 (cells/mL)	2.5 × 10^7^	0.5 × 10^7^	-
Fibroblasts (cells/mL)	-	2.5 × 10^7^	-
THP-1 (cells/mL)	-	0.5 × 10^7^	-

## Data Availability

The raw data supporting the conclusions of this article will be made available by the authors on request.

## Supplementary Materials

The following supporting information can be downloaded at: https://www.mdpi.com/article/10.3390/ijms26125543/s1.

## Abbreviations

The following abbreviations are used in this manuscript:

2D	two-dimensional
3D	three-dimensional
ACEII	angiotensin-converting enzyme 2
ALI	air–liquid interface
CAD	computer-aided design
DAPI	4′,6-diamidino-2-phenylindole
DMEM	Dulbecco’s modified Eagle’s medium
ELISA	enzyme-linked immunoabsorbent assay
GE/mL	genome equivalents per milliliter
HA	hyaluronic acid
IAV	influenza A virus
IAV-NP	IAV nucleoprotein
IL-29	Interleukin 29
Lam-521	laminin-521
NEAA	non-essential amino acids
P/S	penicillin/streptomycin
pan-CK	pan-cytokeratin
PC	phase contrast
pfu	plaque-forming units
PMA	phorbol 12-myristate-13-acetate
PMS	phenazine methosulphate
SARS-CoV-2	severe acute respiratory syndrome coronavirus 2
TEER	transepithelial electrical resistance
XTT	tetrazolium hydroxide salt

## References

[1] Cohen M., Levine S.M., Zar H.J. (2022). World Lung Day: Impact of “the big 5 lung diseases” in the context of COVID-19. Am. J. Physiol. Lung Cell. Mol. Physiol..

[2] Peck K.M., Lauring A.S. (2018). Complexities of Viral Mutation Rates. J. Virol..

[3] Sanjuán R., Nebot M.R., Chirico N., Mansky L.M., Belshaw R. (2010). Belshaw, Viral Mutation Rates. J. Virol..

[4] Chakraborty C., Bhattacharya M., Dhama K. (2023). SARS-CoV-2 Vaccines, Vaccine Development Technologies, and Significant Efforts in Vaccine Development during the Pandemic: The Lessons Learned Might Help to Fight against the Next Pandemic. Vaccines.

[5] Fiolet T., Kherabi Y., MacDonald C.-J., Ghosn J., Peiffer-Smadja N. (2022). Comparing COVID-19 vaccines for their characteristics, efficacy and effectiveness against SARS-CoV-2 and variants of concern: A narrative review. Clin. Microbiol. Infect..

[6] Rijsbergen L.C., van Dijk L.L.A., Engel M.F.M., de Vries R.D., de Swart R.L. (2021). In Vitro Modelling of Respiratory Virus Infections in Human Airway Epithelial Cells—A Systematic Review. Front. Immunol..

[7] Knudsen L., Ochs M. (2018). The micromechanics of lung alveoli: Structure and function of surfactant and tissue components. Histochem. Cell Biol..

[8] Guillot L., Nathan N., Tabary O., Thouvenin G., Le Rouzic P., Corvol H., Amselem S., Clement A. (2013). Alveolar epithelial cells: Master regulators of lung homeostasis. Int. J. Biochem. Cell Biol..

[9] Bouvier N.M., Lowen A.C. (2010). Animal Models for Influenza Virus Pathogenesis and Transmission. Viruses.

[10] Van Norman G.A. (2019). Limitations of Animal Studies for Predicting Toxicity in Clinical Trials: Is it Time to Rethink Our Current Approach?. JACC Basic Transl. Sci..

[11] Wong C.H., Siah K.W., Lo A.W. (2019). Estimation of clinical trial success rates and related parameters. Biostatistics.

[12] Siramshetty V.B., Nickel J., Omieczynski C., Gohlke B.-O., Drwal M.N., Preissner R. (2016). WITHDRAWN—A resource for withdrawn and discontinued drugs. Nucleic Acids Res..

[13] Weidner C., Steinfath M., Opitz E., Oelgeschläger M., Schönfelder G. (2016). Defining the optimal animal model for translational research using gene set enrichment analysis. EMBO Mol. Med..

[14] Hwang K.S., Seo E.U., Choi N., Kim J., Kim H.N. (2022). 3D engineered tissue models for studying human-specific infectious viral diseases. Bioact. Mater..

[15] Melo B.A.G., Benincasa J.C., Cruz E.M., Maricato J.T., Porcionatto M.A. (2020). 3D culture models to study SARS-CoV-2 infectivity and antiviral candidates: From spheroids to bioprinting. Biomed. J..

[16] Weinhart M., Hocke A., Hippenstiel S., Kurreck J., Hedtrich S. (2019). 3D organ models-Revolution in pharmacological research?. Pharmacol. Res..

[17] Zscheppang K., Berg J., Hedtrich S., Verheyen L., Wagner D.E., Suttorp N., Hippenstiel S., Hocke A.C. (2018). Human Pulmonary 3D Models For Translational Research. Biotechnol. J..

[18] Bejoy A.M., Makkithaya K.N., Hunakunti B.B., Hegde A., Krishnamurthy K., Sarkar A., Lobo C.F., Keshav D.V.S., Dharshini G., Dharshini S.D. (2021). An insight on advances and applications of 3d bioprinting: A review. Bioprinting.

[19] Berg J., Weber Z., Fechler-Bitteti M., Hocke A.C., Hippenstiel S., Elomaa L., Weinhart M., Kurreck J. (2021). Bioprinted Multi-Cell Type Lung Model for the Study of Viral Inhibitors. Viruses.

[20] Berg J., Hiller T., Kissner M.S., Qazi T.H., Duda G.N., Hocke A.C., Hippenstiel S., Elomaa L., Weinhart M., Fahrenson C. (2018). Optimization of cell-laden bioinks for 3D bioprinting and efficient infection with influenza A virus. Sci. Rep..

[21] Ali A.S.M., Berg J., Roehrs V., Wu D., Hackethal J., Braeuning A., Woelken L., Rauh C., Kurreck J. (2024). Xeno-Free 3D Bioprinted Liver Model for Hepatotoxicity Assessment. Int. J. Mol. Sci..

[22] Wu D., Berg J., Arlt B., Röhrs V., Al-Zeer M.A., Deubzer H.E., Kurreck J. (2021). Bioprinted Cancer Model of Neuroblastoma in a Renal Microenvironment as an Efficiently Applicable Drug Testing Platform. Int. J. Mol. Sci..

[23] Wu D., Pang S., Berg J., Mei Y., Ali A.S.M., Röhrs V., Tolksdorf B., Hagenbuchner J., Ausserlechner M.J., Deubzer H.E. (2024). Bioprinting of Perfusable Vascularized Organ Models for Drug Development via Sacrificial-Free Direct Ink Writing. Adv. Funct. Mater..

[24] Mahfouzi S.H., Tali S.H.S., Amoabediny G. (2021). 3D bioprinting for lung and tracheal tissue engineering: Criteria, advances, challenges, and future directions. Bioprinting.

[25] Xu J., Wei Y., Zou S., Ye J. (2023). Characterization of 3D-Bioprinted In Vitro Lung Cancer Models Using RNA-Sequencing Techniques. Bioengineering.

[26] Sanchez-Guzman D., Boland S., Brookes O., Mc Cord C., Lai Kuen R., Sirri V., Baeza Squiban A., Devineau S. (2021). Long-Term Evolution of the Epithelial Cell Secretome in Preclinical 3D Models of the Human Bronchial Epithelium. Sci. Rep..

[27] Wang J., Oberley-Deegan R., Wang S., Nikrad M., Funk C.J., Hartshorn K.L., Mason R.J. (2009). Differentiated Human Alveolar Type II Cells Secrete Antiviral IL-29 (IFN-λ1) in Response to Influenza A Infection. J. Immunol..

[28] Wiese-Rischke C., Murkar R.S., Walles H. (2021). Biological Models of the Lower Human Airways—Challenges and Special Requirements of Human 3D Barrier Models for Biomedical Research. Pharmaceutics.

[29] Zou X., Chen K., Zou J., Han P., Hao J., Han Z. (2020). Single-cell RNA-seq data analysis on the receptor ACE2 expression reveals the potential risk of different human organs vulnerable to 2019-nCoV infection. Front. Med..

[30] Chu H., Chan J.F.-W., Yuen T.T.-T., Shuai H., Yuan S., Wang Y., Hu B., Yip C.C.-Y., Tsang J.O.-L., Huang X. (2020). Comparative tropism, replication kinetics, and cell damage profiling of SARS-CoV-2 and SARS-CoV with implications for clinical manifestations, transmissibility, and laboratory studies of COVID-19: An observational study. Lancet Microbe.

[31] Niemeyer D., Mösbauer K., Klein E.M., Sieberg A., Mettelman R.C., Mielech A.M., Dijkman R., Baker S.C., Drosten C., Müller M.A. (2018). The papain-like protease determines a virulence trait that varies among members of the SARS-coronavirus species. PLoS Pathog..

[32] Corman V.M., Landt O., Kaiser M., Molenkamp R., Meijer A., Chu D.K.W., Bleicker T., Brünink S., Schneider J., Schmidt M.L. (2020). Detection of 2019 novel coronavirus (2019-nCoV) by real-time RT-PCR. Euro Surveill..

[33] Are E.B., Song Y., Stockdale J.E., Tupper P., Colijn C. (2023). COVID-19 endgame: From pandemic to endemic? Vaccination, reopening and evolution in low- and high-vaccinated populations. J. Theor. Biol..

[34] Rabaan A.A., Al-Ahmed S.H., Albayat H., Alwarthan S., Alhajri M., Najim M.A., AlShehail B.M., Al-Adsani W., Alghadeer A., Abduljabbar W.A. (2023). Variants of SARS-CoV-2: Influences on the Vaccines’ Effectiveness and Possible Strategies to Overcome Their Consequences. Medicina.

[35] Matarese G., Cava A.L., Horvath T.L. (2012). In vivo veritas, in vitro artificial. Trends Mol. Med..

[36] Berkhout B. (2009). Toward a durable anti-HIV gene therapy based on RNA interference. Ann. N. Y. Acad. Sci..

[37] Stein E.A., Pinkert S., Becher P.M., Geisler A., Zeichhardt H., Klopfleisch R., Poller W., Tschöpe C., Lassner D., Fechner H. (2015). Combination of RNA interference and virus receptor trap exerts additive antiviral activity in coxsackievirus B3-induced myocarditis in mice. J. Infect. Dis..

[38] Schaar K., Geisler A., Kraus M., Pinkert S., Pryshliak M., Spencer J.F., Tollefson A.E., Ying B., Kurreck J., Wold W.S. (2017). Anti-adenoviral Artificial MicroRNAs Expressed from AAV9 Vectors Inhibit Human Adenovirus Infection in Immunosuppressed Syrian Hamsters. Mol. Ther. Nucleic Acids.

[39] Pryshliak M., Hazini A., Knoch K., Dieringer B., Tolksdorf B., Solimena M., Kurreck J., Pinkert S., Fechner H. (2020). MiR-375-mediated suppression of engineered coxsackievirus B3 in pancreatic cells. FEBS Lett..

[40] Tolksdorf B., Heinze J., Niemeyer D., Röhrs V., Berg J., Drosten C., Kurreck J. (2024). Development of a highly stable, active small interfering RNA with broad activity against SARS-CoV viruses. Antiviral Res..

[41] Tolksdorf B., Nie C., Niemeyer D., Röhrs V., Berg J., Lauster D., Adler J.M., Haag R., Trimpert J., Kaufer B. (2021). Inhibition of SARS-CoV-2 Replication by a Small Interfering RNA Targeting the Leader Sequence. Viruses.

[42] Yang J., Petitjean S.J.L., Koehler M., Zhang Q., Dumitru A.C., Chen W., Derclaye S., Vincent S.P., Soumillion P., Alsteens D. (2020). Molecular interaction and inhibition of SARS-CoV-2 binding to the ACE2 receptor. Nat. Commun..

[43] Gard A.L., Luu R.J., Miller C.R., Maloney R., Cain B.P., Marr E.E., Burns D.M., Gaibler R., Mulhern T.J., Wong C.A. (2021). High-throughput human primary cell-based airway model for evaluating influenza, coronavirus, or other respiratory viruses in vitro. Sci. Rep..

[44] Zarkoob H., Allué-Guardia A., Chen Y.-C., Garcia-Vilanova A., Jung O., Coon S., Song M.J., Park J.-G., Oladunni F., Miller J. (2022). Modeling SARS-CoV-2 and influenza infections and antiviral treatments in human lung epithelial tissue equivalents. Commun. Biol..

[45] Sen C., Rickabaugh T.M., Jeyachandran A.V., Yuen C., Ghannam M., Durra A., Aziz A., Castillo K., Garcia G., Purkayastha A. (2025). Optimization of a micro-scale air-liquid-interface model of human proximal airway epithelium for moderate throughput drug screening for SARS-CoV-2. Respir. Res..

[46] Goh K.J., Lu H., Tan E.K., Lee Z.Y., Wong A., Tran T., Dunn N.R., Roy S. (2024). Differentiation of CD166-positive hPSC-derived lung progenitors into airway epithelial cells. Biol. Open.

[47] Jensen C., Teng Y. (2020). Is It Time to Start Transitioning From 2D to 3D Cell Culture?. Front. Mol. Biosci..

[48] Berg J., Kurreck J. (2021). Clean bioprinting—Fabrication of 3D organ models devoid of animal components. ALTEX.

[49] Daniels M.J., Selgrade M.K., Doerfler D., Gilmour M.I. (2003). Kinetic profile of influenza virus infection in three rat strains. Comp. Med..

[50] Li B., Tang Q., Cheng D., Qin C., Xie F.Y., Wei Q., Xu J., Liu Y., Zheng B.-J., Woodle M.C. (2005). Using siRNA in prophylactic and therapeutic regimens against SARS coronavirus in Rhesus macaque. Nat. Med..

[51] Idris A., Davis A., Supramaniam A., Acharya D., Kelly G., Tayyar Y., West N., Zhang P., McMillan C.L.D., Soemardy C. (2021). A SARS-CoV-2 targeted siRNA-nanoparticle therapy for COVID-19. Mol. Ther..

[52] Geisler A., Dieringer B., Elsner L., Klingel K., Klopfleisch R., Vornlocher H.-P., Kurreck J., Fechner H. (2023). Lipid nanoparticle-encapsulated, chemically modified anti-adenoviral siRNAs inhibit hepatic adenovirus infection in immunosuppressed Syrian hamsters. Mol. Ther. Nucleic Acids.

[53] Sitia G., Aiolfi R., Di Lucia P., Mainetti M., Fiocchi A., Mingozzi F., Esposito A., Ruggeri Z.M., Chisari F.V., Iannacone M. (2012). Antiplatelet therapy prevents hepatocellular carcinoma and improves survival in a mouse model of chronic hepatitis B. Proc. Natl. Acad. Sci. USA.

[54] Yant S.R., Mulato A., Hansen D., Tse W.C., Niedziela-Majka A., Zhang J.R., Stepan G.J., Jin D., Wong M.H., Perreira J.M. (2019). A highly potent long-acting small-molecule HIV-1 capsid inhibitor with efficacy in a humanized mouse model. Nat. Med..

[55] Novak N., Weighardt H., Valdelvira R., Izquierdo E., Förster I., Cabanillas B. (2021). Herpes simplex virus 1 proteins can induce skin inflammation in an atopic dermatitis-like mouse model. Exp. Dermatol..

[56] Hassan A.O., Case J.B., Winkler E.S., Thackray L.B., Kafai N.M., Bailey A.L., McCune B.T., Fox J.M., Chen R.E., Alsoussi W.B. (2020). A SARS-CoV-2 Infection Model in Mice Demonstrates Protection by Neutralizing Antibodies. Cell.

[57] Sun J., Zhuang Z., Zheng J., Li K., Wong R.L.-Y., Liu D., Huang J., He J., Zhu A., Zhao J. (2020). Generation of a Broadly Useful Model for COVID-19 Pathogenesis, Vaccination, and Treatment. Cell.

[58] Moysidou C.-M., Barberio C., Owens R.M. (2021). Advances in Engineering Human Tissue Models. Front. Bioeng. Biotechnol..

[59] Karamchand L., Makeiff D., Gao Y., Azyat K., Serpe M.J., Kulka M. (2023). Biomaterial inks and bioinks for fabricating 3D biomimetic lung tissue: A delicate balancing act between biocompatibility and mechanical printability. Bioprinting.

[60] Zimmerling A., Chen X. (2020). Bioprinting for combating infectious diseases. Bioprinting.

[61] Zimmerling A., Dahlan N.A., Zhou Y., Chen X. (2024). Recent frontiers in biofabrication for respiratory tissue engineering. Bioprinting.

[62] Wang Z., Lam E.H.Y., Yu F., Zhu S. (2023). 3D Bioprinting for Next-Generation Personalized Medicine. Int. J. Mol. Sci..

[63] Nam K.-H., Smith A.S.T., Lone S., Kwon S., Kim D.-H. (2015). Biomimetic 3D Tissue Models for Advanced High-Throughput Drug Screening. J. Lab. Autom..

[64] Jensen G., Morrill C., Huang Y. (2018). 3D tissue engineering, an emerging technique for pharmaceutical research. Acta Pharm. Sin. B.

[65] Youk J., Kim T., Evans K.V., Jeong Y.-I., Hur Y., Hong S.P., Kim J.H., Yi K., Kim S.Y., Na K.J. (2020). Three-Dimensional Human Alveolar Stem Cell Culture Models Reveal Infection Response to SARS-CoV-2. Cell Stem Cell.

[66] Salahudeen A.A., Choi S.S., Rustagi A., Zhu J., Van Unen V., De La O S.M., Flynn R.A., Margalef-Català M., Santos A.J.M., Ju J. (2020). Progenitor identification and SARS-CoV-2 infection in human distal lung organoids. Nature.

[67] Si L., Bai H., Rodas M., Cao W., Oh C.Y., Jiang A., Moller R., Hoagland D., Oishi K., Horiuchi S. (2021). A human-airway-on-a-chip for the rapid identification of candidate antiviral therapeutics and prophylactics. Nat. Biomed. Eng..

[68] Lee Y., Lee M.K., Lee H.-R., Kim B., Kim M., Jung S. (2024). 3D-printed airway model as a platform for SARS-CoV-2 infection and antiviral drug testing. Biomaterials.

[69] Zhou J., Li C., Sachs N., Chiu M.C., Wong B.H.-Y., Chu H., Poon V.K.-M., Wang D., Zhao X., Wen L. (2018). Differentiated human airway organoids to assess infectivity of emerging influenza virus. Proc. Natl. Acad. Sci. USA.

[70] Rosellini A., Freer G., Quaranta P., Dovere V., Menichini M., Maggi F., Mazzetti P., Pistello M. (2019). Enhanced in vitro virus expression using 3-dimensional cell culture spheroids for infection. J. Virol. Methods.

[71] Kronemberger G.S., Carneiro F.A., Rezende D.F., Baptista L.S. (2021). Spheroids and organoids as humanized 3D scaffold-free engineered tissues for SARS-CoV-2 viral infection and drug screening. Artif. Organs.

[72] Sasaki M., Kishimoto M., Itakura Y., Tabata K., Intaruck K., Uemura K., Toba S., Sanaki T., Sato A., Hall W.W. (2021). Air-liquid interphase culture confers SARS-CoV-2 susceptibility to A549 alveolar epithelial cells. Biochem. Biophys. Res. Commun..

[73] Park B.K., Kim D., Park S., Maharjan S., Kim J., Choi J.-K., Akauliya M., Lee Y., Kwon H.-J. (2021). Differential Signaling and Virus Production in Calu-3 Cells and Vero Cells upon SARS-CoV-2 Infection. Biomol. Ther..

[74] Gandikota C., Vaddadi K., Sivasami P., Huang C., Liang Y., Pushparaj S., Deng X., Channappanava R., Metcalf J.P., Liu L. (2024). The use of human iPSC-derived alveolar organoids to explore SARS-CoV-2 variant infections and host responses. J. Med. Virol..

[75] Fisher C.R., Medie F.M., Luu R.J., Gaibler R.B., Mulhern T.J., Miller C.R., Zhang C.J., Rubio L.D., Marr E.E., Vijayakumar V. (2023). A High-Throughput, High-Containment Human Primary Epithelial Airway Organ-on-Chip Platform for SARS-CoV-2 Therapeutic Screening. Cells.

[76] Flerlage T., Boyd D.F., Meliopoulos V., Thomas P.G., Schultz-Cherry S. (2021). Influenza virus and SARS-CoV-2: Pathogenesis and host responses in the respiratory tract. Nat. Rev. Microbiol..

[77] Taubenberger J.K., Morens D.M. (2008). The Pathology of Influenza Virus Infections. Annu. Rev. Pathol. Mech. Dis..

[78] Chen A., Dong J., Yuan X., Bo H., Li S., Wang C., Duan Z., Zheng L. (2019). Anti-H7N9 avian influenza A virus activity of interferon in pseudostratified human airway epithelium cell cultures. Virol. J..

[79] Yeo M., Sarkar A., Singh Y.P., Derman I.D., Datta P., Ozbolat I.T. (2024). Synergistic coupling between 3D bioprinting and vascularization strategies. Biofabrication.

[80] Weinheimer V.K., Becher A., Tönnies M., Holland G., Knepper J., Bauer T.T., Schneider P., Neudecker J., Rückert J.C., Szymanski K. (2012). Influenza A Viruses Target Type II Pneumocytes in the Human Lung. J. Infect. Dis..

[81] Niemeyer D., Stenzel S., Veith T., Schroeder S., Friedmann K., Weege F., Trimpert J., Heinze J., Richter A., Jansen J. (2022). SARS-CoV-2 Variant Alpha Has a Spike-Dependent Replication Advantage over the Ancestral B.1 Strain in Human Cells with Low ACE2 Expression. PLOS Biol..

[82] Michi A.N., Proud D. (2021). A toolbox for studying respiratory viral infections using air-liquid interface cultures of human airway epithelial cells. Am. J. Physiol.-Lung Cell. Mol. Physiol..

[83] Wu N.-H., Yang W., Beineke A., Dijkman R., Matrosovich M., Baumgärtner W., Thiel V., Valentin-Weigand P., Meng F., Herrler G. (2016). The differentiated airway epithelium infected by influenza viruses maintains the barrier function despite a dramatic loss of ciliated cells. Sci. Rep..

