# Supplementary material for: Bioprinted Four-Cell-Type Lung Model for Viral Infection Studies Under Air–Liquid Interface Conditions
Source: Int J Mol Sci. 2025 Jun 10;26(12):5543. doi: 10.3390/ijms26125543 (PMC12193617; doi:10.3390/ijms26125543)
Supplement: Supplementary file 1 [file ijms-26-05543-s001.zip › ijms-3647836-supplementary.pdf]

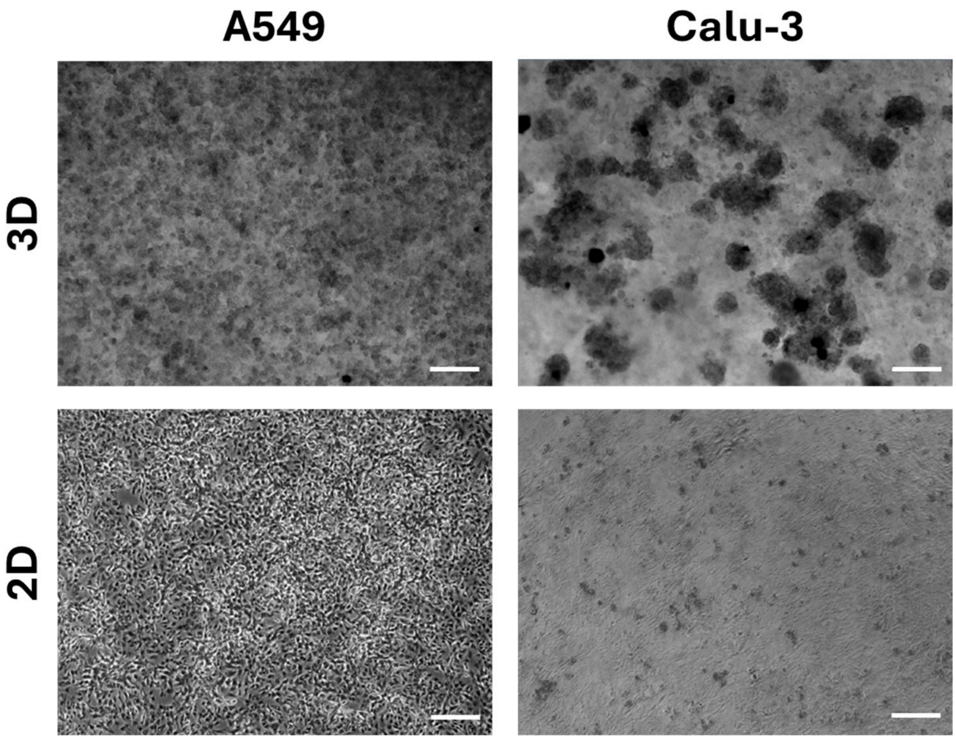

Figure S1. Morphology of A549 and Calu-3 cells in 2D and 3D lung models. For 2D cultivation A549 and Calu-3 cells were seeded into 24-well plates and analyzed 48 h after seeding by phase contrast microscopy. For 3D cultivation the improved bioprinted multi-cell type lung model was seeded with A549 cells or Calu-3 cells. Seven days after the start of ALI cultivation, the cells were analyzed by phase contrast microscopy. Scale bar: 200 µm.
